# A novel web-based calculator to predict 30-day all-cause in-hospital mortality for 7,202 elderly patients with heart failure in ICUs: a multicenter retrospective cohort study in the United States

**DOI:** 10.3389/fmed.2023.1237229

**Published:** 2023-09-15

**Authors:** Zhongjian Wang, Jian Huang, Yang Zhang, Xiaozhu Liu, Tingting Shu, Minjie Duan, Haolin Wang, Chengliang Yin, Junyi Cao

**Affiliations:** ^1^Artificial Intelligence Laboratory, Pharnexcloud Digital Technology (Chengdu) Co. Ltd., Chengdu, China; ^2^Graduate School, Guangxi University of Chinese Medicine, Nanning, China; ^3^College of Medical Informatics, Chongqing Medical University, Chongqing, China; ^4^Medical Data Science Academy, Chongqing Medical University, Chongqing, China; ^5^Department of Cardiology, Daping Hospital, The Third Military Medical University (Army Medical University), Chongqing, China; ^6^Faculty of Medicine, Macau University of Science and Technology, Macau, Macau SAR, China; ^7^Department of Medical Quality Control, The First People's Hospital of Zigong City, Zigong, China

**Keywords:** big data, web-based, calculator, heart failure, death, elderly

## Abstract

**Background and aims:**

Heart failure (HF) is a significant cause of in-hospital mortality, especially for the elderly admitted to intensive care units (ICUs). This study aimed to develop a web-based calculator to predict 30-day in-hospital mortality for elderly patients with HF in the ICU and found a relationship between risk factors and the predicted probability of death.

**Methods and results:**

Data (*N* = 4450) from the MIMIC-III/IV database were used for model training and internal testing. Data (*N* = 2,752) from the eICU-CRD database were used for external validation. The Brier score and area under the curve (AUC) were employed for the assessment of the proposed nomogram. Restrictive cubic splines (RCSs) found the cutoff values of variables. The smooth curve showed the relationship between the variables and the predicted probability of death. A total of 7,202 elderly patients with HF were included in the study, of which 1,212 died. Multivariate logistic regression analysis showed that 30-day mortality of HF patients in ICU was significantly associated with heart rate (HR), 24-h urine output (24h UOP), serum calcium, blood urea nitrogen (BUN), NT-proBNP, SpO_2_, systolic blood pressure (SBP), and temperature (*P* < 0.01). The AUC and Brier score of the nomogram were 0.71 (0.67, 0.75) and 0.12 (0.11, 0.15) in the testing set and 0.73 (0.70, 0.75), 0.13 (0.12, 0.15), 0.65 (0.62, 0.68), and 0.13 (0.12, 0.13) in the external validation set, respectively. The RCS plot showed that the cutoff values of variables were HR of 96 bmp, 24h UOP of 1.2 L, serum calcium of 8.7 mg/dL, BUN of 30 mg/dL, NT-pro-BNP of 5121 pg/mL, SpO_2_ of 93%, SBP of 137 mmHg, and a temperature of 36.4°C.

**Conclusion:**

Decreased temperature, decreased SpO_2_, decreased 24h UOP, increased NT-proBNP, increased serum BUN, increased or decreased SBP, fast HR, and increased or decreased serum calcium increase the predicted probability of death. The web-based nomogram developed in this study showed good performance in predicting 30-day in-hospital mortality for elderly HF patients in the ICU.

## Introduction

Heart failure (HF) is a typical cardiovascular syndrome of aging and a leading cause of morbidity and mortality. It is mainly caused by common cardiovascular diseases in the elderly and age-related changes in cardiovascular structure and function. The incidence of HF reaches 39.8% for the adult population aged over 80 years and 27.1% for patients who are 65–79 years old (1). HF patients in the ICU were reported to have high in-hospital mortality (2), especially for the elderly (3). Previous studies have reported the risk factors for morality in HF patients (4–6). However, the evidence of risk factors for 30-day in-hospital mortality in elderly patients with HF during ICU admission is insufficient.

Recently, the nomogram was widely adopted as an accurate tool to evaluate the prognosis in HF patients (7, 8). However, a convenient and effective web-based prediction model for elderly HF patients admitted to the ICU remains lacking. Therefore, this study aimed to establish a web-based nomogram to predict the 30-day in-hospital mortality for elderly HF patients during ICU admission and discover the potential risk factors, which could support clinical decision-making.

## Methods

### Data source

We conducted a retrospective cohort study with de-identified clinical data extracted from the Medical Information Mart for Intensive Care III/IV (MIMIC-III/IV) database and eICU-CRD (certification ID: 42039823) (9). The MIMIC-III is a publicly available single-center critical care database that contains de-identified clinical data for over 50,000 patients admitted to the Beth Israel Deaconess Medical Center. The MIMIC-IV database is a publicly available critical care database containing de-identified clinical data that contains over 70,000 ICU admissions across the United States from 2008 to 2019. The eICU-CRD database is a publicly available multi-center critical care database that was made available by Philips Healthcare in partnership with the MIT Laboratory for Computational Physiology and contains over 200,000 patients admitted to the ICU from 2014 to 2015. Additionally, the model was then externally validated in patients enrolled in the eICU-CRD database.

### Study population and outcome

Patients diagnosed with HF and over the age of 65 in their last ICU stay were enrolled in the study cohort. HF was identified based on the International Classification of Diseases-Ninth Revision (ICD-9/10) diagnosis codes. Only first-time ICU admission patients were included in this study. The exclusion criteria for this study include (1) patients aged <65 years; (2) patients without a record of N-terminal pro-brain natriuretic peptide (NT-proBNP); (3) patients without a record of an electrocardiogram; and (4) missing more than 30% of patient data. The primary outcome was 30-day in-hospital mortality.

### Data extraction and preprocessing

Clinical data on the participants were collected from the three databases based on clinical guidelines and other literature published previously on relevant topics (5). Baseline characteristics with 35 variables for the included patients were recorded on the first admission to the hospital within 24 h, including vital signs, demographics, laboratory tests, and comorbidities.

### Statistical analysis

Normally distributed continuous variables were expressed as the median with a standard error. Non-normally distributed continuous variables were expressed as the median with an interquartile range (IQR). Categorical data were expressed as frequency (percentage, %) and compared using the chi-square test. A Mann–Whitney U-test was applied to compare non-normally distributed variables. Missing values are imputed by the K-nearest neighbor (KNN) algorithm.

The study cohort was divided randomly into a training set with 80% of patients from MIMIC III/IV for developing the prediction model and a testing set with 20% of patients for evaluating the performance of the model. Additionally, the model was then externally validated in patients enrolled in the eICU-CRD database.

Univariate analysis was performed on the clinical data of patients who died and those who survived. The differences were statistically significant and were further included in the LASSO regression analysis. The variables selected by LASSO were included in the multivariate logistic regression analysis. The LASSO analysis of elderly patients with HF for 30-day in-hospital mortality was conducted to adjust potential confounding factors by regularization. This method can improve the prediction accuracy and dimensionality reduction of high-dimensional data. Variables with non-zero coefficients in the LASSO regression with 10-fold cross-validation were selected for further analysis to ensure minimal autocorrelation. The web-based nomogram, a web calculator, can accurately estimate the risk probability of clinical outcomes based on individual characteristics (7). Then, this study developed a nomogram for the retained variables with non-zero coefficients in LASSO regression and multivariable logistic regression in the training set. The Brier score and area under the curve (AUC) were employed for the predictive performance of the dynamic nomogram. A restrictive cubic spline was applied to find the cutoff value of the variable. The smooth curve was used to show the relationship between variables and the predicted probability of death. The patients in the training set were divided into the ultra-senior group (aged over 80 years) and the elderly group (65 ≤ age ≤ 80 years) for subgroup analysis. The calibration curve was applied to analyze the agreement between the predictive results and actual observations. Clinical usefulness was evaluated by decision curve analysis (DCA) and the clinical impact curve (CIC).

All statistical analyses were performed with R 4.1.3 and RStudio 2022.02.3. The R package “DMwR2” was used for KNN algorithm imputation. The LASSO and logistic regression analyses were performed by the R package “glmnet”. The R package “DynNom” was used for the dynamic nomogram. The R package “riskRegression” was used for calculating Brier scores. The receiver operating characteristic curve analysis was conducted using the “ROCR” package. A *P*-value of < 0.05 was considered statistically significant.

## Results

### Patients' characteristics

**Figure 1:**
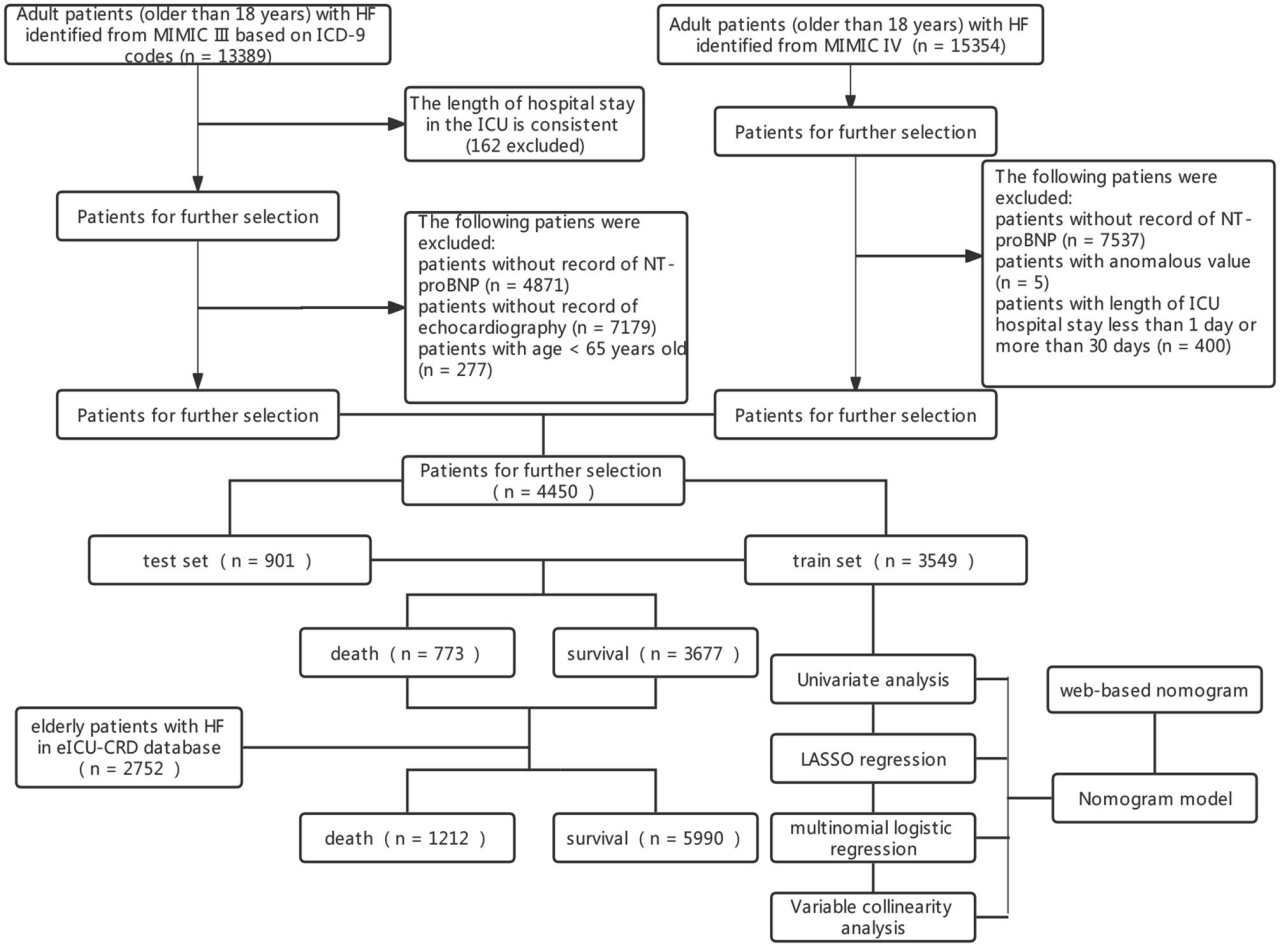
Screening process of included patients and the constructed model. HF, heart failure; ICUs, intensive care units; MIMIC-III, Medical Information Mart for Intensive Care III; ICD, International Classification of Diseases.

A total of 7,202 elderly patients with HF were enrolled from the three databases with ICU admissions. The screening process is shown in Figure 1. This study divided 4,450 patients from MIMIC III/IV into the training set (3,549 participants) and the testing set (901 participants). The characteristics of those patients are shown in Table 1. A total of 2,752 patients from the eICU-CRD database were analyzed as an external validation set (Supplementary Table 1). In total, the median age of HF patients in this study was 80 years old, and 1,979 (46%) patients were women. There were 1,785 (40%) patients with hypertension, 2,386 (54%) with AF, 1,420 (32%) with diabetes, 2,049 (46%) with hyperlipidemia, and 1,485 (33%) with COPD. During hospital admission, 617 (17%) HF patients died within 30 days in the training set, 156 (17%) in the testing set, and 439 (16.0%) in the external validation set.

**Table 1:** Characteristics of elderly patients with heart failure from MIMIC III/IV.

**Variables**	**Total (*N =* 4,450)**	**Testing set (*N =* 901)**	**Training set (*N =* 3,549)**	***p***
Age, Median (Q1, Q3)	80 (72, 86)	80 (72, 86)	80 (72, 87)	0.863
**Gender, n (%)**				0.619
Female	1,979 (46)	411 (46)	1,639 (46)	
Male	2,471 (54)	490 (54)	1,910 (54)	
**Hypertension, n (%)**				0.945
No	2,665 (60)	541 (60)	2,124 (60)	
Yes	1,785 (40)	360 (40)	1,425 (40)	
**Atrial fibrillation, n (%)**				1
No	2,064 (46)	418 (46)	1,646 (46)	
Yes	2,386 (54)	483 (54)	1,903 (54)	
**Diabetes, n (%)**				0.337
No	3,030 (68)	601 (67)	2,429 (68)	
Yes	1,420 (32)	300 (33)	1,120 (32)	
**Hyperlipemia, n (%)**				0.273
No	2,401 (54)	471 (52)	1,930 (54)	
Yes	2,049 (46)	430 (48)	1,619 (46)	
**COPD, n (%)**				0.702
No	2,965 (67)	595 (66)	2,370 (67)	
Yes	1,485 (33)	306 (34)	1,179 (33)	
HR, Median (Q1, Q3)	96 (82.13, 113)	95 (82, 112)	96.38 (82.41, 113)	0.586
SBP, Median (Q1, Q3)	137 (121, 154)	137 (120.32, 153)	137.05 (121, 155)	0.496
DBP, Median (Q1, Q3)	78 (63.41, 94)	77.13 (63.39, 94)	78 (63.41, 94)	0.872
RR, Median (Q1, Q3)	27 (22.39, 32)	26 (22, 31)	27 (22.65, 32)	0.034
Temperature, Median (Q1, Q3)	36.44 (36.13, 36.67)	36.44 (36.13, 36.67)	36.44 (36.13, 36.67)	0.368
SpO_2_, Median (Q1, Q3)	93 (90, 95.46)	93 (90, 95.19)	93 (90, 95.48)	0.853
24h UOP, Median (Q1, Q3)	1.27 (0.83, 1.81)	1.24 (0.8, 1.82)	1.28 (0.85, 1.81)	0.183
Hematocrit, Median (Q1, Q3)	33.7 (29.27, 38.15)	33.7 (29.27, 38.4)	33.7 (29.28, 38.1)	0.765
RBC, Median (Q1, Q3)	3.71 (3.24, 4.22)	3.71 (3.25, 4.23)	3.71 (3.23, 4.22)	0.572
MCH, Median (Q1, Q3)	29.8 (28.1, 31.39)	29.7 (28.2, 31.2)	29.8 (28.1, 31.4)	0.625
MCHC, Median (Q1, Q3)	32.5 (31.3, 33.5)	32.6 (31.4, 33.61)	32.5 (31.3, 33.5)	0.074
MCV, Median (Q1, Q3)	91.18 (87, 96)	91 (87, 95.17)	91.3 (87, 96)	0.245
RDW, Median (Q1, Q3)	15.17 (14.1, 16.71)	15.03 (14, 16.7)	15.2 (14.1, 16.76)	0.162
WBC, Median (Q1, Q3)	9.7 (7.3, 13.3)	9.9 (7.3, 13.4)	9.7 (7.3, 13.2)	0.362
platelet, Median (Q1, Q3)	216 (162, 283)	218 (167, 284)	215 (161, 283)	0.201
PT, Median (Q1, Q3)	14.04 (12.4, 18.29)	13.9 (12.4, 18.5)	14.1 (12.4, 18.15)	0.651
INR, Median (Q1, Q3)	1.28 (1.1, 1.7)	1.23 (1.1, 1.7)	1.3 (1.1, 1.7)	0.577
NT-proBNP, Median (Q1, Q3)	4,994 (1,954, 1,2455)	4,698 (2,000, 12,327)	5,025 (1,926, 12,488)	0.563
Creatinine, Median (Q1, Q3)	1.3 (0.97, 1.9)	1.3 (0.94, 1.84)	1.3 (0.99, 1.9)	0.819
BUN, Median (Q1, Q3)	30 (20.89, 45.62)	30 (20, 46.25)	29.78 (21, 45)	0.896
Glucose, Median (Q1, Q3)	136 (111, 174)	139 (110.33, 178)	135 (111, 173)	0.269
Potassium, Median (Q1, Q3)	4.3 (3.94, 4.8)	4.3 (3.99, 4.8)	4.3 (3.93, 4.8)	0.321
Sodium, Median (Q1, Q3)	138.78 (135.92, 141)	138.25 (135, 141)	138.92 (136, 141)	0.327
Calcium, Median (Q1, Q3)	8.77 (8.4, 9.06)	8.8 (8.4, 9.04)	8.76 (8.4, 9.07)	0.979
chloride, Median (Q1, Q3)	101 (97, 104.12)	101 (97, 104)	101 (97.33, 104.17)	0.195
AP, Median (Q1, Q3)	15 (13, 18)	15.3 (13, 18)	15 (13, 18)	0.331
Bicarbonate, Median (Q1, Q3)	25 (22, 28)	25 (22, 28.43)	25 (22, 28)	0.944
**Death, n (%)**				0.999
No	3,677 (83)	745 (83)	2,932 (83)	
Yes	773 (17)	156 (17)	617 (17)	

### Predictors' selection

**Figure 2:**
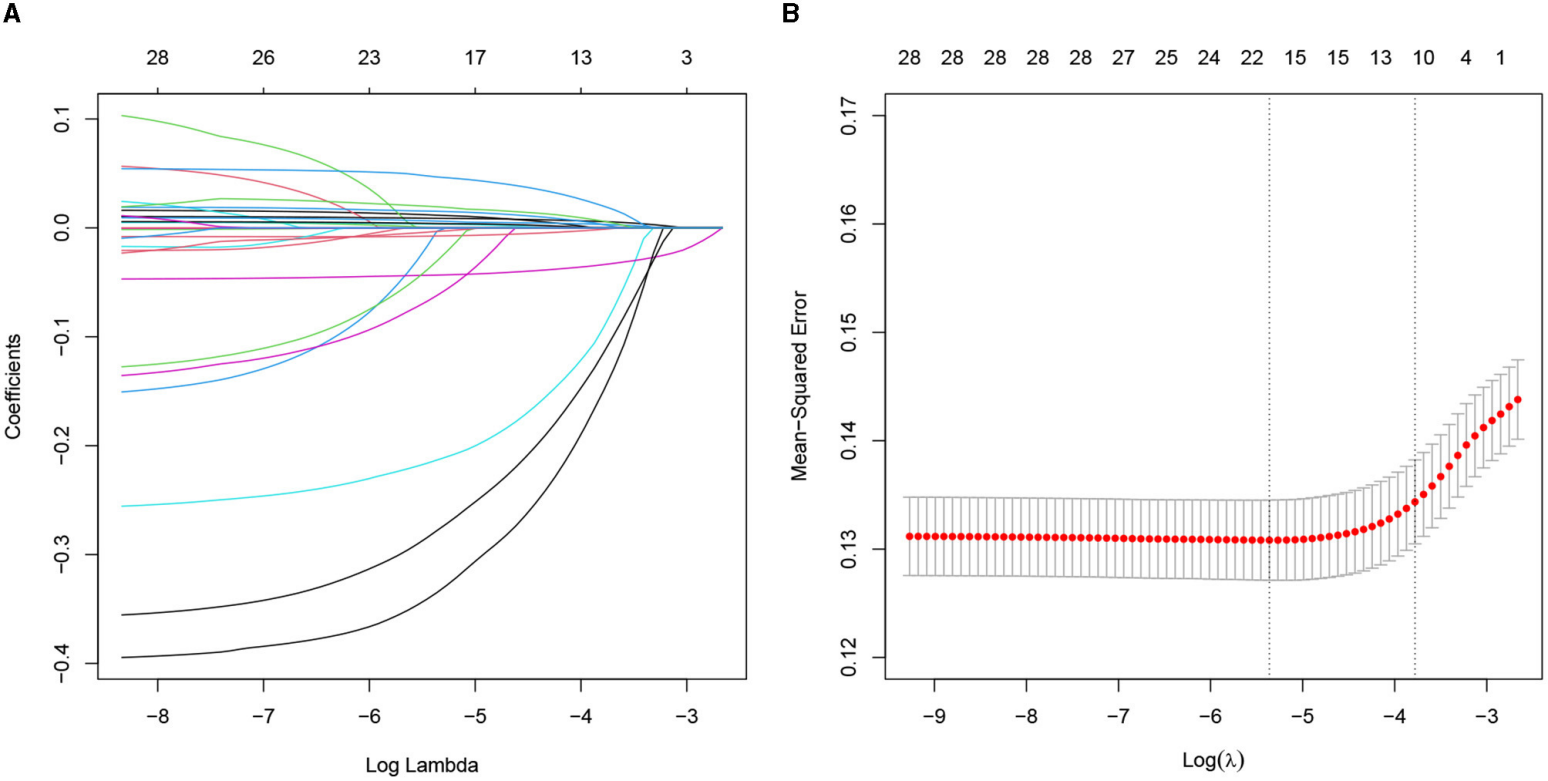
Predictors selected by the LASSO method. **(A)** The coefficients of all predictors gradually return to zeros through 10-fold cross-validation. **(B)** Eleven predictors with non-zero coefficients at the rightmost dashed line. LASSO, least absolute shrinkage and selection operator.

The univariate analysis of the variables is shown in Supplementary Table 2, which was included in the LASSO regression analysis. The penalty on the β-coefficients was controlled by the tuning parameter λ (λ = 0.0228) in the LASSO regression with 10-fold cross-validation (Figure 2). All the selected variables were analyzed by multivariate logistic regression (Table 2). The independent prediction factors associated with a 30-day mortality of elderly HF patients in ICU admission were heart rate [odds ratio (OR) = 1.38, 95% confidence interval (CI): 1.11–1.77, *P* < 0.01], SBP (OR = 0.64, 95% CI: 0.51–0.81, *P* < 0.01), temperature (OR = 0.89, 95% CI: 0.79–0.99, *P* < 0.01), SpO_2_ (OR = 0.74, 95% CI: 0.64–0.85, *P* < 0.01), 24h UOP (OR = 0.67, 95% CI: 0.54–0.82, *P* < 0.01), NT-proBNP (OR = 1.12, 95% CI: 1.00–1.24, *P* < 0.01), BUN (OR = 1.36, 95% CI: 1.16–1.60, *P* < 0.01), and serum calcium (OR = 0.73, 95% CI: 0.61–0.86, *P* < 0.01).

**Table 2:** The multivariate logistic regression of selected variables by LASSO regression.

	**Coef**	**S.E**.	**Wald Z**	**OR(95%CI)**	**Pr(>|Z|)**
Intercept	13.5993	4.2948	3.17		0.0015
HR	0.0105	0.0036	2.9	1.38 (1.11,1.72)	0.0037
SBP	−0.0131	0.0035	−3.7	0.64 (0.51,0.82)	0.0002
RR	0.0202	0.0126	1.61	1.21 (0.96,1.52)	0.1083
Temperature	−0.2133	0.1086	−1.96	0.89 (0.79,0.99)	0.0495
SpO_2_	−0.0559	0.0131	−4.28	0.74 (0.64,0.85)	<0.0001
24h UOP	−0.3169	0.0823	−3.85	0.67 (0.54,0.82)	0.0001
RDW	0.0272	0.0307	0.89	1.08 (0.92,1.26)	0.375
NT-proBNP	0	0	1.97	1.12 (1.00,1.24)	0.0486
BUN	0.0128	0.0033	3.89	1.36 (1.17,1.60)	0.0001
Calcium	−0.3766	0.1022	−3.68	0.73 (0.61,0.86)	0.0002
AP	0.0233	0.0207	1.13	1.12 (0.92,1.38)	0.26

### Nomogram construction and validation

**Figure 3:**
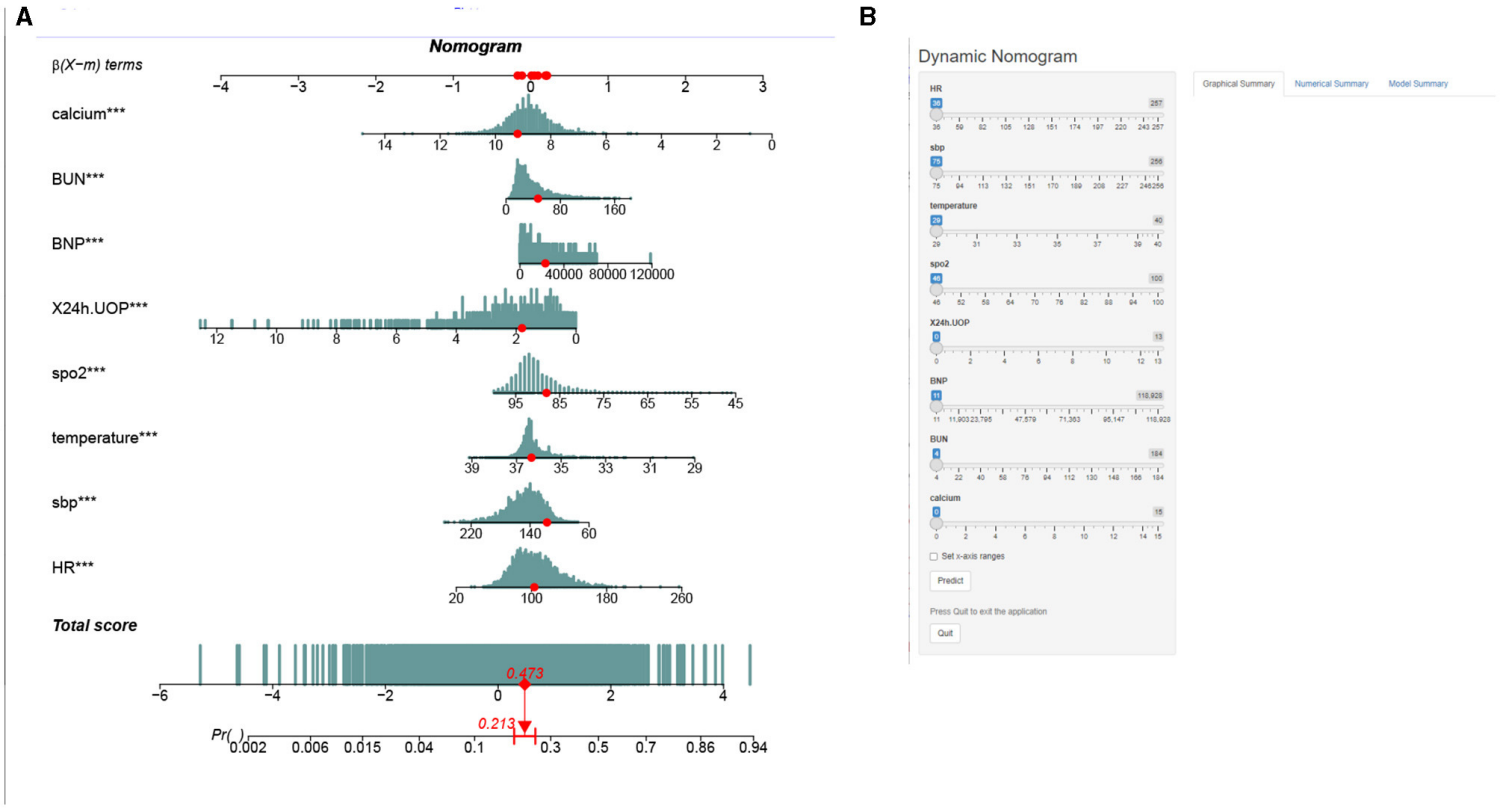
Nomogram to estimate the risk of mortality in elderly with heart failure. The blue histogram in **(A)** represents the distribution of variables, and the red dots represent specific values. **(B)** An interface display of the web-based nomogram. We describe the value score for each corresponding parameter and the probability of mortality for a specific instance. The numbers “1” and “0” in renal failure and deficiency anemias mean “No” and “Yes”, respectively. Urine output, urine output in first 24 h. BUN, serum blood urea nitrogen; INR, international normalized ratio.

**Figure 4:**
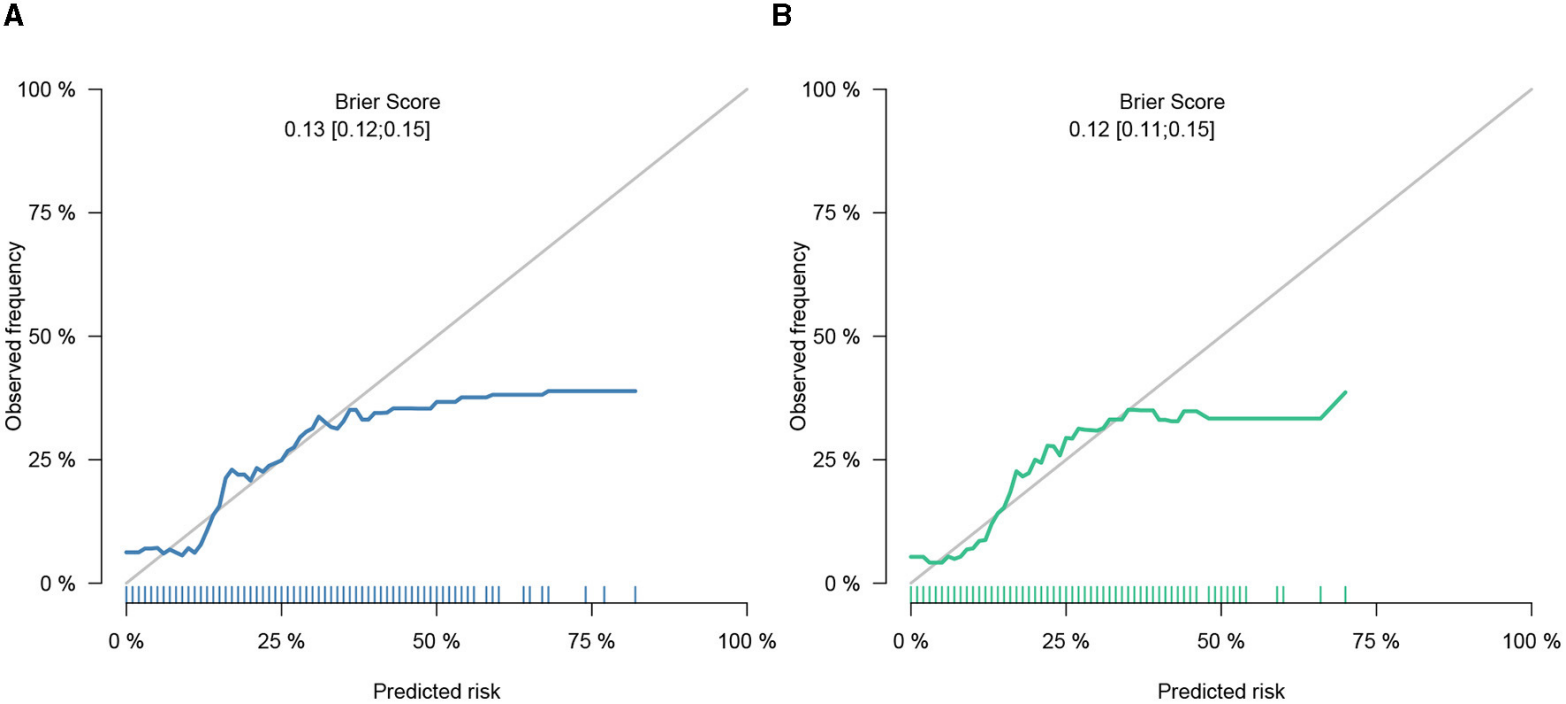
Calibration of nomogram in the training set and the testing set. **(A)** Calibration belt in the training set did not cross the diagonal bisector line. **(B)** Calibration belt in the testing set did not cross the diagonal bisector line. Those suggesting that the prediction models had a strong concordance performance. The calibration curve demonstrated superior agreement between the estimated and actual observations.

**Figure 5:**
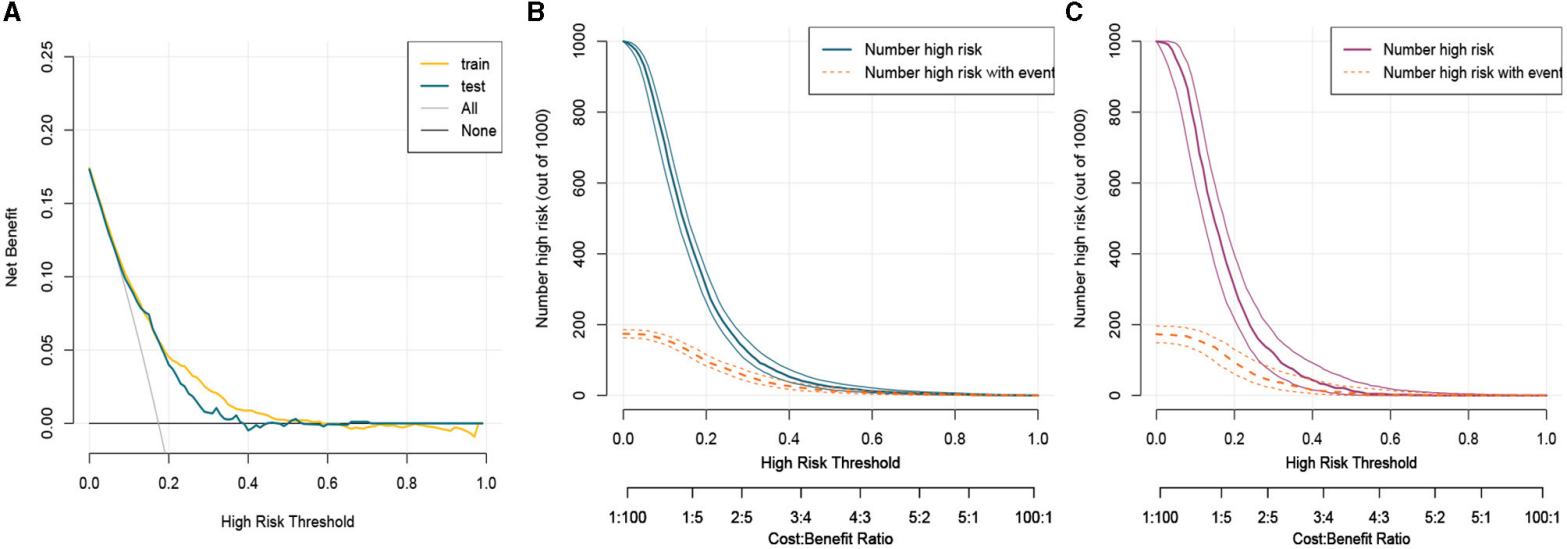
Clinical usefulness of nomogram in the training set and the testing set. **(A)** Decision curve analysis curve in the training set and the testing set. **(B)** Clinical impact curve in the training set. **(C)** Clinical impact curve in the testing set. CIC and DCA showed that the nomogram had a superior overall net benefit within the wide and practical ranges of threshold probabilities and impacted clinical outcomes. Train, train; test, test.

**Figure 6:**
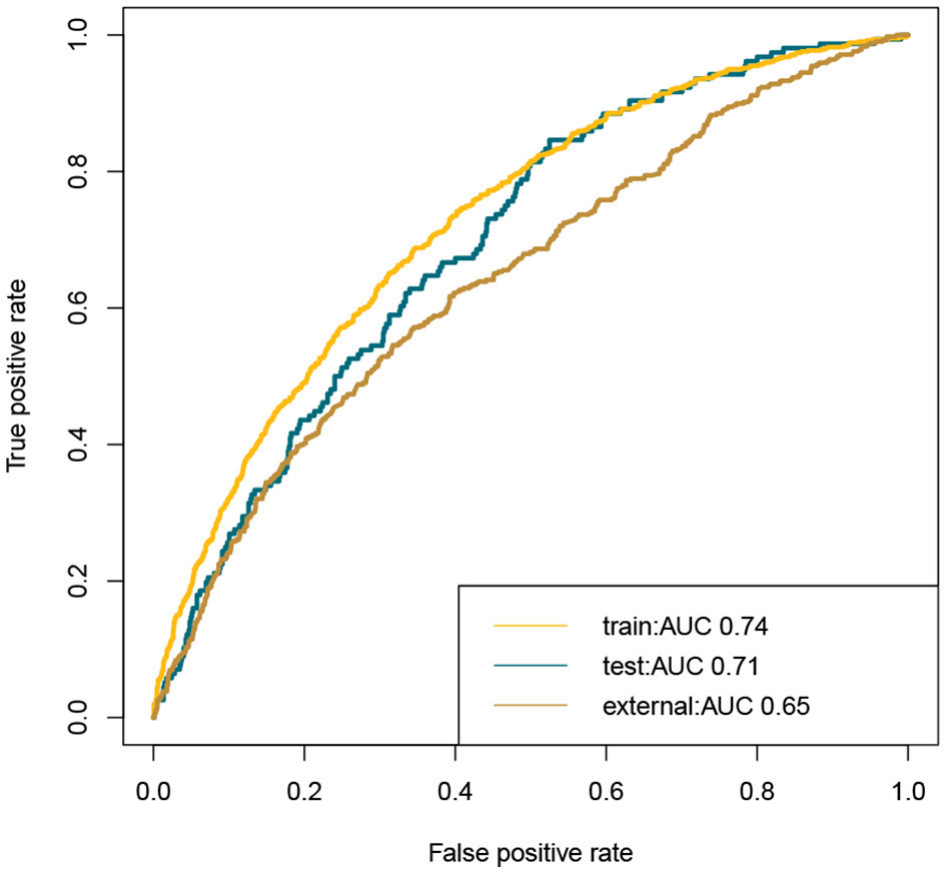
ROC plot of nomogram in the training set, testing set, and external validation set. The AUC showed favorable accuracy of the nomogram model. Train, training set; test, testing set; ROC, receiver operating characteristic curves; AUROC, area under the receiver operating characteristic curves.

**Figure 7:**
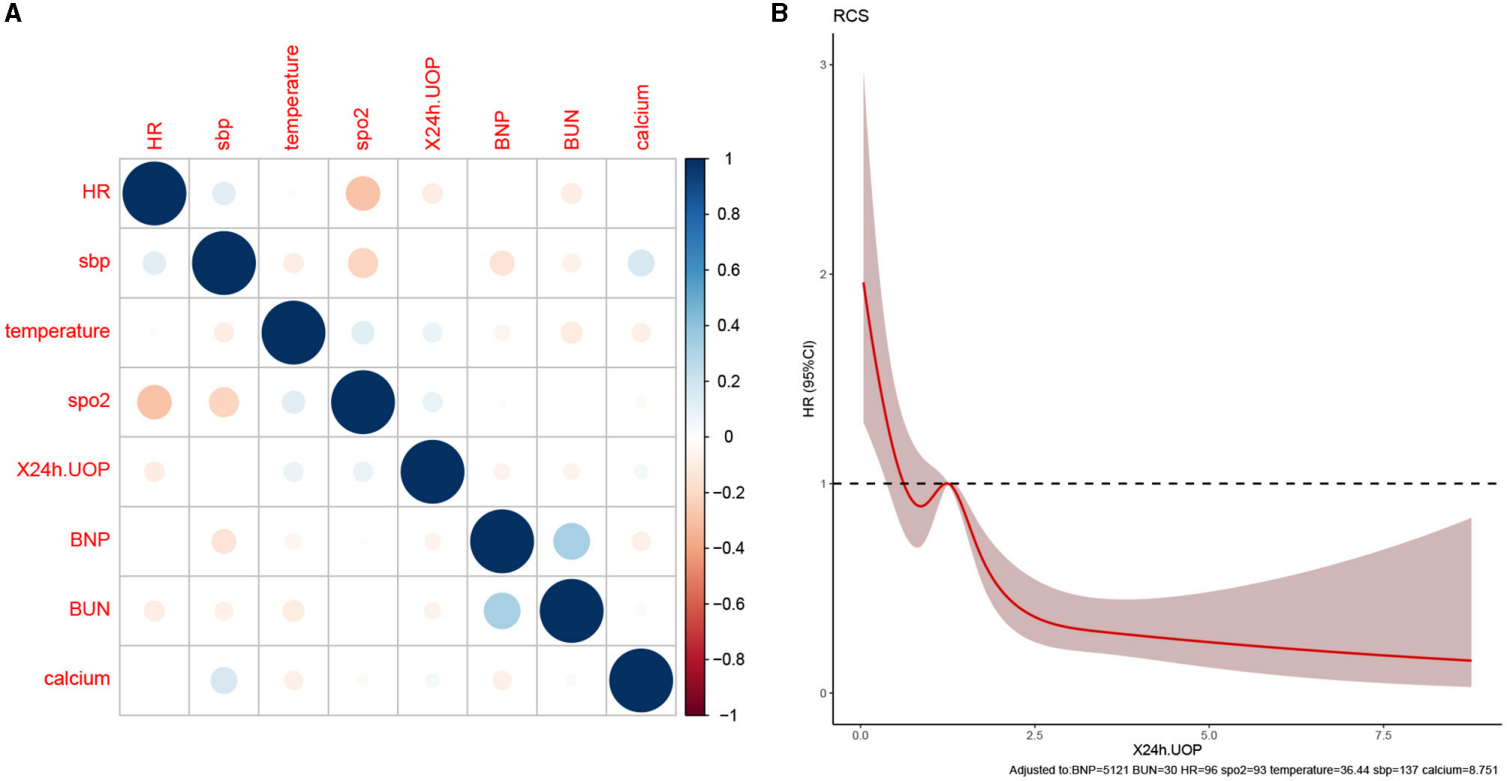
Variable collinearity analysis and RCS plot. **(A)** The correlation coefficient between variables; blue represents a positive correlation, red represents a negative correlation, and the darker the color, the stronger the correlation. **(B)** RCS plot shows the size of the cutoff value of the variable, the intersection of the red solid line, and the dotted line is the cutoff value for 24 h UOP.

This study developed a practiced eight-factor web-based nomogram based on the analysis of MLR in the training set (Figure 3). The AUC of the nomogram model was 0.71 (95% CI: 0.67, 0.75) in the testing set, 0.73 (95% CI: 0.70, 0.75) in the training set, and 0.65 (95% CI: 0.62, 0.68) in the external validation set. The values of the Brier score of the nomogram model were 0.12 (95%CI: 0.11, 0.15) in the testing set, 0.13 (95%CI: 0.12, 0.15) in the training set, and 0.13 (95%CI: 0.12, 0.13) in the external validation set. The calibration curve demonstrated that the nomogram adequately fit both in the training set and in the testing set (Figure 4). The DCA and CIC showed the net benefit and threshold probability of nomograms in the training set and the testing set (Figure 5). The ROC plot demonstrated the predictive probability of the nomogram in the training set, testing set, and external validation set, with AUCs of 0.74, 0.71, and 0.65, respectively (Figure 6). The variable correlation diagram is shown in Figure 7A. The VIF values and tolerance values of the variables are shown in Table 3.

**Table 3:** Variance inflation factor and tolerance values for variables.

**Term**	**VIF**	**Increased SE**	**Tolerance**
HR	1.23	1.11	0.81
SBP	1.21	1.1	0.83
Temperature	1.07	1.04	0.93
SpO_2_	1.36	1.16	0.74
24 h UOP	1.04	1.02	0.96
NT-proBNP	1.12	1.06	0.89
BUN	1.17	1.08	0.86
Calcium	1.04	1.02	0.96

### Variables and death prediction probability analysis

**Figure 8:**
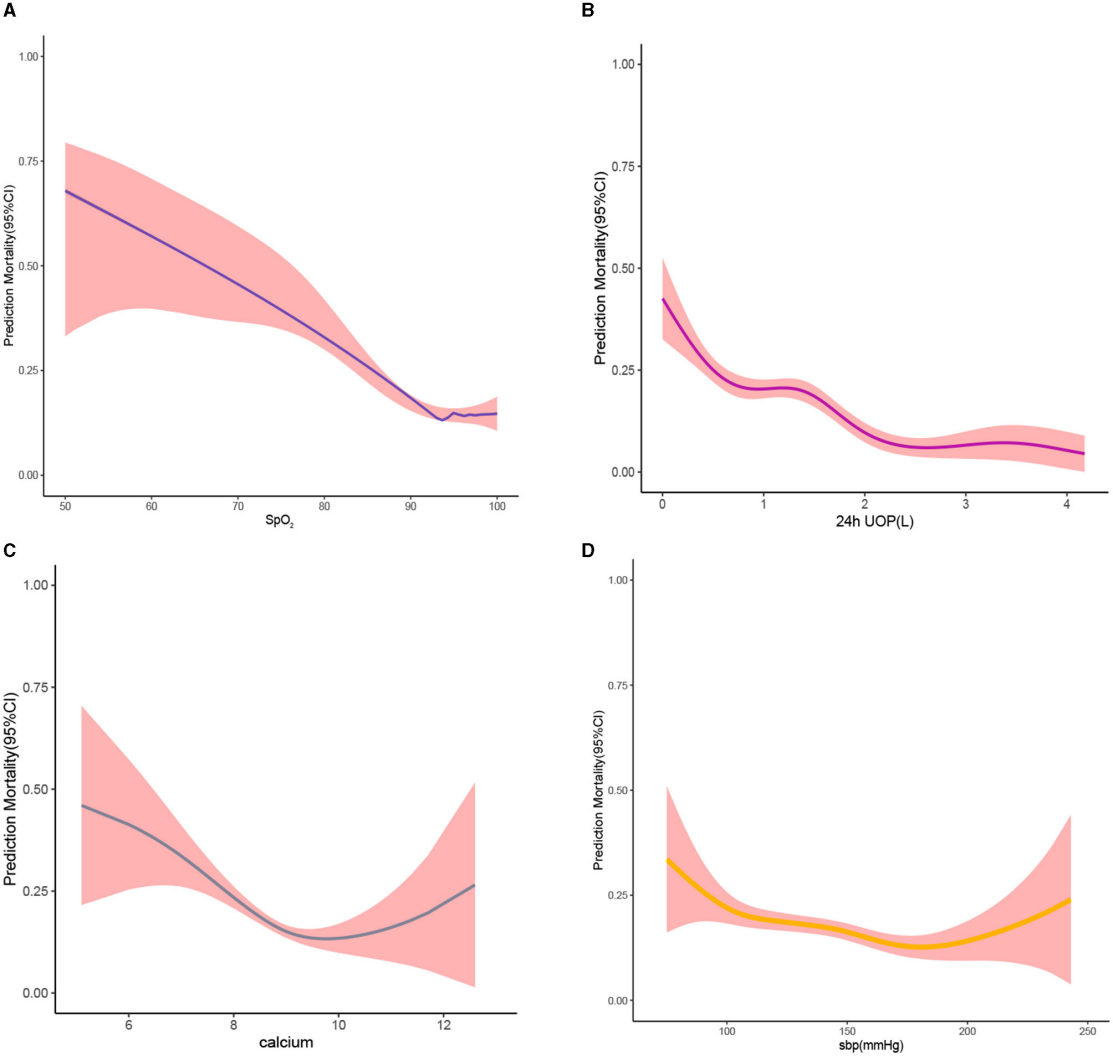
Smooth curve. **(A)** For the relationship between blood oxygen saturation and predicted probability of death; the larger the value of blood oxygen saturation, the lower the predicted probability of death. **(B)** For the relationship between 24h UOP and predicted probability of death; the larger the value of 24h UOP, the higher the predicted probability of death small. **(C)** The relationship between serum calcium levels and predicted probability of death. **(D)** The relationship between systolic blood pressure and predicted probability of death. Red-filled shading represents confidence intervals.

The RCS plot showed that the cutoff value of variables were HR of 96 bmp, SBP of 137 mmHg, temperature of 36.4°C, SpO_2_ of 93%, 24h UOP of 1.2L, NT-proBNP of 5121 pg/dL, BUN of 30 mg/dL, and serum calcium of 8.7 mg/dL (Figures 7B, 8). The smoothed curve (Figure 8) showed the values of SpO_2_ (93%), serum calcium (10 mg/dL), 24h UOP (<1L), and SBP (100 to 140 mmHg) with the lowest probability of death. However, the predicted probability of death changed dramatically when the SBP was <100 mmHg or more than 160 mmHg.

### Subgroup analysis

The results of the subgroup analysis (Table 4) showed that, in the subgroup of ultra-elderly patients aged > 80 years, all the selected variables except temperature were risk factors for 30-day mortality during ICU admission (*P* < 0.05). In another subgroup (65 ≤ age ≤ 80), all the variables were significant risk factors (*P* < 0.05).

**Table 4:** Subgroup analysis for age.

**Subgroup**	**Variables**	**OR**	**Low CI**	**Upper CI**	***P*-Value**
**65** ≤ **age** ≤ **80**					
	HR	1.01	1.01	1.02	<0.001
	SBP	0.99	0.98	1	0.001
	Temperature	0.66	0.54	0.79	<0.001
	SpO_2_	0.95	0.93	0.97	<0.001
	X24h.UOP	0.6	0.49	0.72	<0.001
	NT-proBNP	1	1	1	0.008
	BUN	1.01	1	1.01	0.012
	Calcium	0.7	0.58	0.86	0.001
**Age** > **80**					
	HR	1.01	1.01	1.02	<0.001
	SBP	0.99	0.98	1	0.001
	Temperature	0.89	0.73	1.09	0.256
	SpO_2_	0.95	0.93	0.96	<0.001
	X24h.UOP	0.76	0.65	0.88	<0.001
	NT-proBNP	1	1	1	0.007
	BUN	1.01	1.01	1.02	<0.001
	Calcium	0.67	0.54	0.83	<0.001

## Discussion

This study was the first attempt to develop a convenient and effective web-based nomogram model for predicting the 30-day mortality of elderly HF patients in the MIMIC-III/IV database and the eICU-CRD database. Our analysis showed that 30-day mortality during ICU admission was significantly associated with heart rate, SBP, temperature, SpO_2_, 24h UOP, NT-proBNP, BUN, and serum calcium. The nomogram model produced in this study showed excellent performance. The RCS plot and the smoothed curves increased clinical practicality with the cutoff value of variables and the value of variables with the lowest probability of death.

The incidence and prevalence of HF progressively increase in parallel with the population's age. HF remains one of the most immediate causes of death in the United States as well as in most other countries in the world (10). One study analyzed the outcomes of 1,767 HF patients from 4 countries, in which 387 patients died and 343 were hospitalized for exacerbation during the follow-up duration, and a gradual decline in survival was observed for both mortality and hospitalization over 3 years (1). A key difficulty in the management of elderly HF patients is to identify the risk factor for early mortality, as the clinical situation is often unpredictable. Previous studies have shown that sex, blood pressure, age, serum sodium, BUN, NT-proBNP levels, and EF were risk factors for mortality in HF patients (3, 11–13). Our study identified risk factors for hospital mortality for elderly HF patients during ICU admission, which is crucial for evaluating the severity of illness and initiating targeted treatment.

There are some points that should be highlighted in this study. First, our nomogram model included a large number of patients from three databases and was designed specifically for elderly patients with HF during ICU admission, unlike other score models that were designed for all ICU patients. Second, with the analysis of the RCS plot and the smoothed curves, it can be utilized not only to predict the risk of mortality but also to identify the cutoff value of variables and the value of variables with the lowest probability of death, which will have a significant impact on the physician's decision-making. Third, our nomogram showed an excellent predictive probability in the external validation set, illustrating a strong degree of credibility. Additionally, we used three databases to produce an optimal prognostic prediction model for 30-day mortality and discovered general variables as risk factors, including heart rate, SBP, temperature, SpO_2_, 24h UOP, NT-proBNP, BUN, and serum calcium, which were accessible for physicians' clinical practices. Finally, LASSO regression with a satisfied penalty condition was applied to improve the prediction accuracy and was suitable for reduction in high-dimensional data, which penalized the coefficients of some useless variable features and enhanced the generalization ability of the model.

Previous studies proved that increased heart rate represents an important indicator of mortality, with each acceleration of the heart rate over 70 bpm increasing the risk not only in patients with cardiovascular disease but also in the general population (14). Heart rate was qualified as a modifiable risk factor for heart failure. In our study, we found that a heart rate of 96 bmp in elderly HF patients is the cutoff value for the risk of mortality. The better the heart rate is controlled, the lower the risk of death for the old. Williamson et al. (15) reported that adults aged 75 years or older with an SBP of <120 mmHg had significantly lower rates of fatal and non-fatal major cardiovascular events and death from any cause. Elderly patients with HF are a special group accompanied by cardiac insufficiency. Our study has obtained a more detailed SBP control interval of 100 to 140 mmHg, which is conducive to better regulation of blood pressure in clinical practice. A previous study (16) demonstrated that decreasing temperatures increased HF rehospitalization. Our study found that a decreased temperature of <36.4°C is associated with mortality, which may be associated with systemic failure resulting in peripheral hypoperfusion. Decreased SpO_2_ is often accompanied by respiratory insufficiency, which is something that many HF patients (17), especially the elderly, should focus on. Our study suggests that an increase in SpO_2_ of more than 93% in these patients may have a protective effect. For elderly patients with HF, decreased 24 h UOP or increased NT-proBNP often indicate severe impairment of cardiac function, especially when combined with renal insufficiency (increased serum BUN), leading to an increased risk of death. Lutsey et al. (18) found that high serum calcium was independently associated with a greater risk of incident HF. We found that too low or too high serum calcium had a poor prognosis for elderly HF patients.

Logistic regression models showed a linear relationship between variables and the outcome. However, the relationship between the univariate analysis and outcome may be non-linear in order to find cutoff values for variables and a range of values that indicate danger or protective significance. In this study, the cutoff values of variables were analyzed using RCS, and it was found that the relationship between SpO_2_, temperature, 24 h UOP, calcium, and death risk was non-linear.

The establishment of a prediction model for patients has always been a hot topic (19, 20). Therefore, in this study, the nomogram based on the eight risk factors above was applied to predicting the mortality risk of ICU-admitted older patients with HF. The application of a dynamic nomogram predicted the patient's 30-day probability of in-hospital death in elderly patients with HF. Health professionals could take targeted interventions according to the scores of different risk factors on the nomogram for each subject, improving the efficiency of interventions early. However, a non-professional statistician may find a regular nomogram inconvenient because it requires the risk calculation for each variable. Therefore, we constructed a web-based dynamic nomogram (https://huangjian.shinyapps.io/elderHFDynNomapp/) based on the nomogram model. Clinicians could access the website directly anytime and anywhere and input corresponding predictors to obtain an individual's death probability with a 95% CI. This would undoubtedly simplify the application process and facilitate decision-making, which may help tailor individualized, risk-based treatment strategies. Furthermore, it is convenient for elderly patients with HF and their caregivers to receive targeted interventions based on the results of a dynamic nomogram. This study extends this knowledge in several important ways. First, compared with the convenient logistic regression model, the nomogram produced in this study assessed risk stratification. Second, this nomogram has been generated specifically for elderly HF patients. We have provided an example of a patient in the nomogram using clinical indicators to predict the patient's 30-day probability of in-hospital death (Figure 3). This model predicted a lower probability of death for the patient at 21.3%, and we eventually confirmed that this patient was, indeed, alive when he was discharged from the hospital. The AUC value is >0.7, and the Brier score index value is <0.15, indicating that the model calibration is high and the prediction performance is good. As such, the nomogram model can accurately identify high-risk elderly HF patients and carry out early intervention to prolong survival time.

There were some limitations in this study. First, patients included in this study lacked clinical indicators and outcomes with longer follow-up duration. Multicenter clinical studies with long-term follow-up and large sample sizes are needed to validate this nomogram in future. Second, some risk factors were only analyzed for the first time within 24 h of admission, such as blood pressure, temperature, and heart rate, which were always volatile. The analysis of factor instabilities or timing changes is required for further study.

## Conclusion

This study developed a convenient and effective web-based nomogram tool to predict the 30-day mortality of elderly HF patients in the MIMIC-III/IV and the eICU-CRD databases. Increased HR, extreme SBP, decreased temperature, decreased SpO_2_, decreased 24 h UOP, increased NT-proBNP, increased serum BUN, and extreme serum calcium could increase the probability of death.

## Data availability statement

The original contributions presented in the study are included in the article/Supplementary material, further inquiries can be directed to the corresponding authors.

## Ethics statement

The MIMIC-III/IV and eICU-CRD databases were approved by the Massachusetts Institute of Technology (Cambridge, MA) and Beth Israel Deaconess Medical Center (Boston, MA).

## Author contributions

ZW, YZ, and XL were responsible for conceiving the idea of the study and collecting the data. MD and HW reviewed this manuscript. TS was responsible for the manuscript writing and revising. JH and CY were responsible for the study design, data analysis, and manuscript writing. JC and ZW were responsible for APC. All authors contributed to the article and approved the submitted version.
