# Supplementary material for: A novel web-based calculator to predict 30-day all-cause in-hospital mortality for 7,202 elderly patients with heart failure in ICUs: a multicenter retrospective cohort study in the United States
Source: Front Med (Lausanne). 2023 Sep 15;10:1237229. doi: 10.3389/fmed.2023.1237229 (PMC10541310; doi:10.3389/fmed.2023.1237229)
Supplement: Supplementary file 1 [file Table_1.docx]

Supplement table1 Characteristics of elderly patients with heart failure from eICU-CRD database.

| Variables | Total (n = 2752) | Survival  (n = 2335) | Death  (n = 439) | p |
| --- | --- | --- | --- | --- |
| Age, Median (Q1, Q3) | 78 (71, 85) | 78 (71, 84) | 80 (72, 85) | 0.005 |
| BUN, Median (Q1, Q3) | 29 (20, 45) | 29 (19, 43) | 35 (23, 54) | < 0.001 |
| Calcium, Median (Q1, Q3) | 8.9 (8.5, 9.3) | 8.9 (8.5, 9.3) | 8.8 (8.4, 9.2) | 0.006 |
| Temperature, Median (Q1, Q3) | 37 (37, 97.65) | 37 (37, 97.95) | 37 (37, 39.4) | 0.119 |
| BNP, Median (Q1, Q3) | 1142 (490.88, 3069.25) | 1136 (478, 3091.2) | 1207 (552, 2808) | 0.397 |
| HR, Median (Q1, Q3) | 88 (73, 104) | 88 (73, 103) | 90 (75, 109) | 0.014 |
| SpO_2_, Median (Q1, Q3) | 96 (93, 99) | 96 (93, 99) | 96 (92, 99) | 0.184 |
| SBP, Median (Q1, Q3) | 127 (108, 149) | 129 (109, 151) | 117 (102, 137) | < 0.001 |
| 24h UOP, Median (Q1, Q3) | 1.01 (1.01, 1.02) | 1.01 (1.01, 1.02) | 1.01 (1.01, 1.02) | < 0.001 |

Supplement table 2 The univariate analysis of the variables.

| Variables | Total  (n = 4450) | Survival  (n = 3677) | Death  (n = 773) | p |
| --- | --- | --- | --- | --- |
| Age, Median (Q1, Q3) | 80 (72, 86) | 79 (71, 86) | 81 (74, 87) | < 0.001 |
| Gender, n (%) |  |  |  | 0.005 |
| Female | 2287 (51) | 1901 (51) | 386 (50) |  |
| Male | 2163 (49) | 1776 (49) | 387 (50) |  |
| Hypertension, n (%) |  |  |  | < 0.001 |
| No | 2665 (60) | 2158 (59) | 507 (66) |  |
| Yes | 1785 (40) | 1519 (41) | 266 (34) |  |
| Atrial fibrillation, n (%) |  |  |  | < 0.001 |
| No | 2064 (46) | 1750 (48) | 314 (41) |  |
| Yes | 2386 (54) | 1927 (52) | 459 (59) |  |
| Diabetes, n (%) |  |  |  | 0.049 |
| No | 3030 (68) | 2480 (67) | 550 (71) |  |
| Yes | 1420 (32) | 1197 (33) | 223 (29) |  |
| Hyperlipidemia, n (%) |  |  |  | 0.016 |
| No | 2401 (54) | 1953 (53) | 448 (58) |  |
| Yes | 2049 (46) | 1724 (47) | 325 (42) |  |
| COPD, n (%) |  |  |  | 0.165 |
| No | 2965 (67) | 2467 (67) | 498 (64) |  |
| Yes | 1485 (33) | 1210 (33) | 275 (36) |  |
| HR, Median (Q1, Q3) | 96 (82.13, 113) | 94.12 (81, 111) | 105 (89, 120) | < 0.001 |
| SBP, Median (Q1, Q3) | 137 (121, 154) | 138 (121.39, 155) | 134 (118.12, 150) | < 0.001 |
| DBP, Median (Q1, Q3) | 78 (63.41, 94) | 77.83 (63, 94) | 80 (65, 96) | 0.023 |
| RR, Median (Q1, Q3) | 27 (22.39, 32) | 26 (22, 31) | 29 (24.13, 34) | < 0.001 |
| Temperature, Median (Q1, Q3) | 36.44 (36.13, 36.67) | 36.44 (36.19, 36.67) | 36.39 (35.94, 36.64) | < 0.001 |
| SpO_2_, Median (Q1, Q3) | 93 (90, 95.46) | 93 (90, 95.62) | 91 (87, 95) | < 0.001 |
| 24h UOP, Median (Q1, Q3) | 1.27 (0.83, 1.81) | 1.32 (0.87, 1.93) | 1.05 (0.67, 1.47) | < 0.001 |
| Hematocrit, Median (Q1, Q3) | 33.7 (29.27, 38.15) | 33.76 (29.3, 38.2) | 33.6 (29.1, 37.89) | 0.253 |
| RBC, Median (Q1, Q3) | 3.71 (3.24, 4.22) | 3.73 (3.24, 4.23) | 3.65 (3.19, 4.15) | 0.079 |
| MCH, Median (Q1, Q3) | 29.8 (28.1, 31.39) | 29.8 (28.13, 31.37) | 29.8 (27.8, 31.4) | 0.714 |
| MCHC, Median (Q1, Q3) | 32.5 (31.3, 33.5) | 32.5 (31.4, 33.5) | 32.3 (31, 33.4) | < 0.001 |
| MCV, Median (Q1, Q3) | 91.18 (87, 96) | 91 (87, 96) | 92 (87, 97) | 0.011 |
| RDW, Median (Q1, Q3) | 15.17 (14.1, 16.71) | 15.06 (14, 16.6) | 15.6 (14.4, 17.3) | < 0.001 |
| WBC, Median (Q1, Q3) | 9.7 (7.3, 13.3) | 9.5 (7.2, 12.9) | 11 (7.6, 15.8) | < 0.001 |
| Platelet, Median (Q1, Q3) | 216 (162, 283) | 218 (165.07, 283) | 207 (153, 289) | 0.006 |
| PT, Median (Q1, Q3) | 14.04 (12.4, 18.29) | 13.9 (12.4, 17.6) | 14.8 (12.8, 20.3) | < 0.001 |
| INR, Median (Q1, Q3) | 1.28 (1.1, 1.7) | 1.23 (1.1, 1.6) | 1.35 (1.1, 1.9) | < 0.001 |
| NT-proBNP, Median (Q1, Q3) | 4994 (1954, 12455) | 4692 (1864, 11168) | 7509 (2633, 17863) | < 0.001 |
| Creatinine, Median (Q1, Q3) | 1.3 (0.97, 1.9) | 1.3 (0.95, 1.8) | 1.38 (1, 2.1) | < 0.001 |
| BUN, Median (Q1, Q3) | 30 (20.89, 45.62) | 29 (20, 44) | 35 (23.87, 53) | < 0.001 |
| Glucose, Median (Q1, Q3) | 136 (111, 174) | 136 (111, 173.4) | 135.62 (109.83, 177) | 0.867 |
| Potassium, Median (Q1, Q3) | 4.3 (3.94, 4.8) | 4.3 (3.92, 4.72) | 4.4 (4, 4.9) | < 0.001 |
| Sodium, Median (Q1, Q3) | 138.78 (135.92, 141) | 139 (136, 141) | 138 (135, 141) | < 0.001 |
| Calcium, Median (Q1, Q3) | 8.77 (8.4, 9.06) | 8.8 (8.43, 9.07) | 8.62 (8.24, 9) | < 0.001 |
| Chloride, Median (Q1, Q3) | 101 (97, 104.12) | 101 (98, 104.09) | 100 (96, 104.31) | 0.008 |
| AP, Median (Q1, Q3) | 15 (13, 18) | 15 (13, 17.5) | 16 (13.95, 19) | < 0.001 |
| Bicarbonate, Median (Q1, Q3) | 25 (22, 28) | 25 (22, 28.29) | 24 (21, 27) | < 0.001 |
